# Supplementary material for: Micrometer-Scale Membrane Transition of Supported Lipid Bilayer Membrane Reconstituted with Cytosol of Dictyostelium discoideum
Source: Life (Basel). 2017 Mar 7;7(1):11. doi: 10.3390/life7010011 (PMC5370411; doi:10.3390/life7010011)
Supplement: Supplementary file 1 [file life-07-00011-s001.pdf]

Supplementary Materials: Micrometer-Scale Membrane Transition of Supported Lipid Bilayer Membrane Reconstituted with Cytosol of *Dictyostelium discoideum*

Kei Takahashi and Taro Toyota

Video S1. Video of the pore formation on the POPC SLB membrane after injection of the cytosol extract of PH-crac-RFP/PTEN-GFP co-expressing cells (Figure 4A in main text). The pores on the SLB membrane were remarkably formed for initial 444–552 s after cytosol injection (24–30 s). Movie was generated from phase contrast images taken with a time interval of 1 frame every 6 s. The size of the observation space is 75.50 μm × 75.50 μm.

Video S2. Video of the tubular vesicle generation on the POPC SLB membrane on the flat slide glass (not using bottom open dish) after injection of the cytosol extract of PH-crac-RFP/PTEN-GFP co-expressing cells (Figure S4 in main text). Tubular giant vesicles on the SLB membrane were generated remarkably for initial ~300 s after cytosol injection (24–30 s). Movie was generated from phase contrast images taken with a time interval of 1 frame every 6 s. The size of the observation space is 75.50 μm × 75.50 μm.

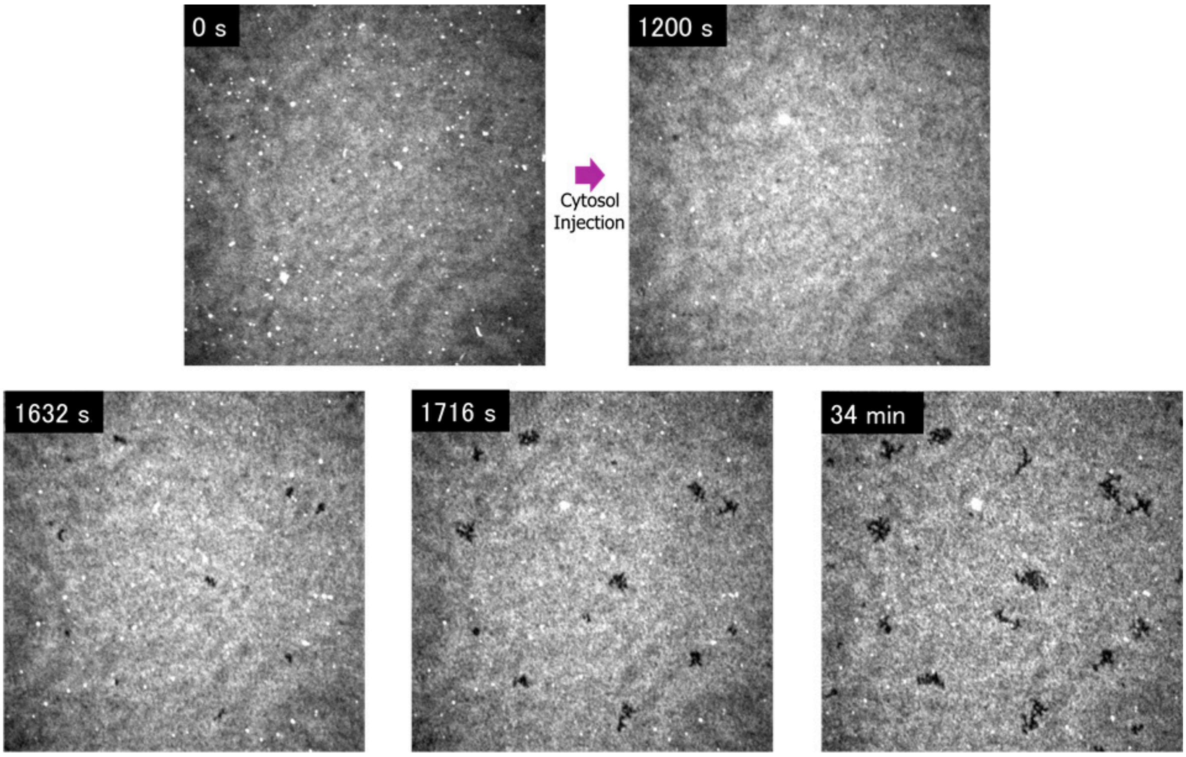

Figure S1. Pore formation on the POPC SLB membrane after injection of the cytosol at low room temperature. Time-course change of microscopy images of the POPC SLB membrane in the presence of the cytosol extract of PH-crac-RFP/PTEN-GFP co-expressing cells at 21 °C was monitored by the confocal laser scanning microscopy. The pores on the SLB membrane were remarkably formed for initial 1632–3000 s after cytosol injection (24–30 s). The size of each observation space is 75.50 μm × 75.50 μm.

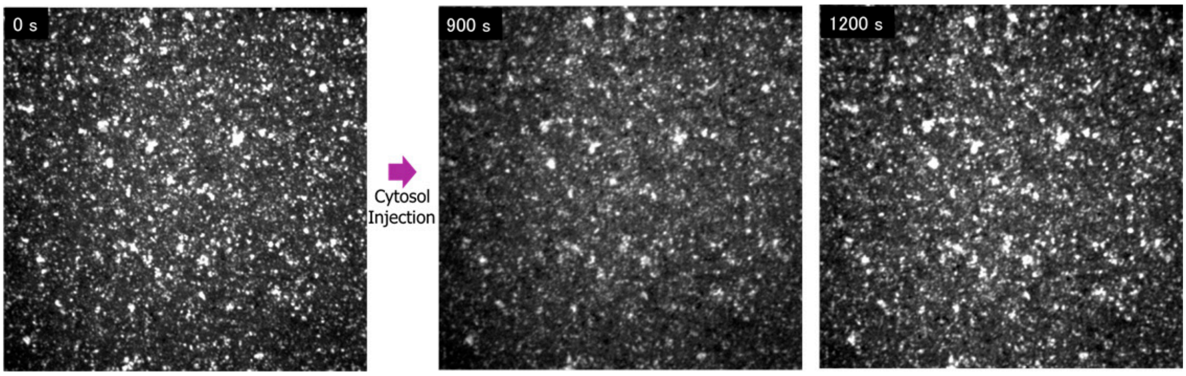

Figure S2. Microscopic observation of the time-course change of POPC/18:0–20:4 PI(3,4,5)P3 SLB membrane. The time of injection of the cytosol extract of PH-crac-RFP/PTEN-GFP co-expressing cells is 24–30 s. The size of each observation space is 75.50 μm × 75.50 μm.

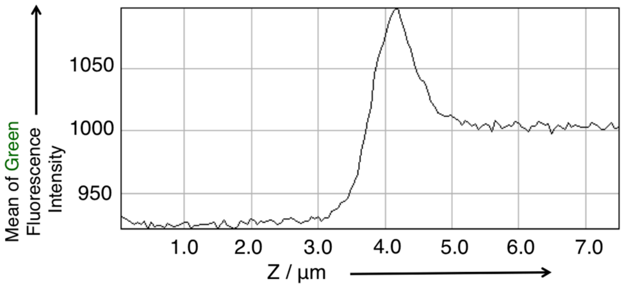

Figure S3. Mean fluorescence intensity profile of green fluorescence (PTEN-GFP) image plotted along the z position of POPC/18:0–20:4 PI(4,5)P2 (2 mol %) SLB membrane after injection of cytosolic extract from PH-crac-RFP/PTEN-GFP co-expressing cells. The size of each image is 75.5 μm × 75.5 μm as shown in Figure 5A.

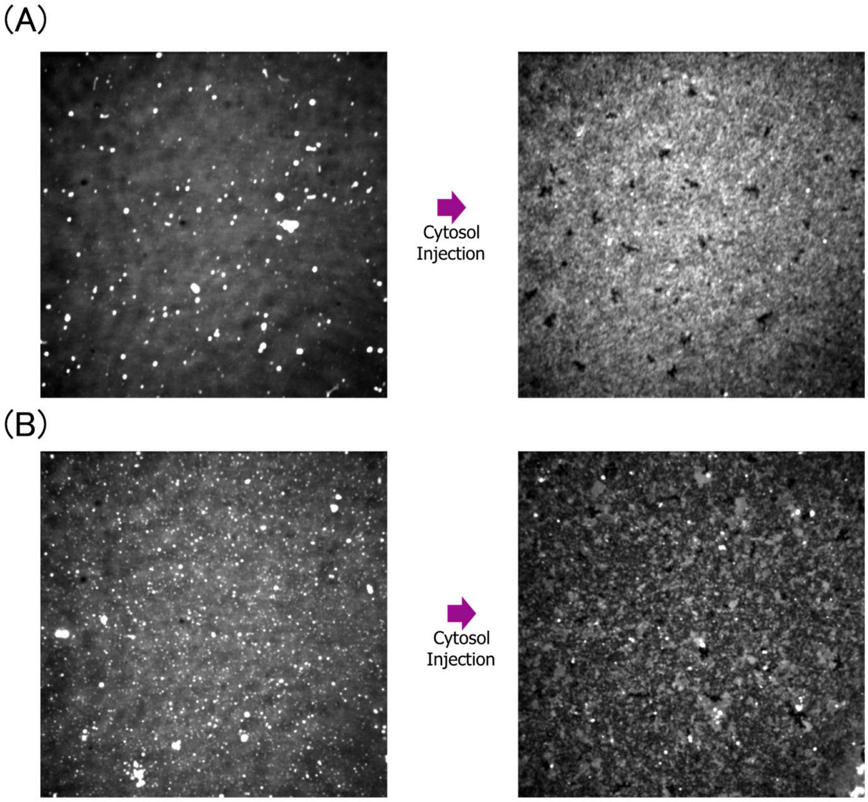

Figure S4. *Cont.*

(C)

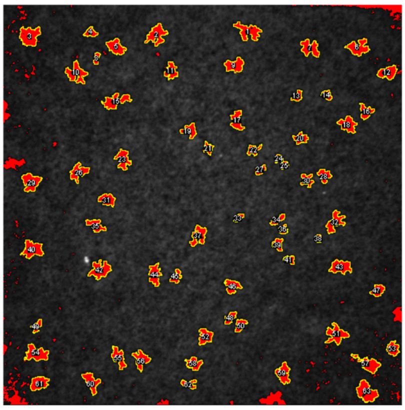

Figure S4. POPC/08:0 PI(4,5)P2 and POPC/08:0 PI(3,4,5)P3 SLB membrane transition after injection of the cytosol extract of PH-crac-RFP/PTEN-GFP co-expressing cells and pore size distribution of SLB membrane in each condition. Fluorescence image of (**A**) POPC/08:0 PI(4,5)P2 and (**B**) POPC/08:0 PI(3,4,5)P3 SLB membrane images before and after injection of the cytosol; The size of each observation space is 75.50 μm × 75.50 μm. (**C**) Area measurement of pores formed at the POPC SLB membrane Figure 4 A by ImageJ.

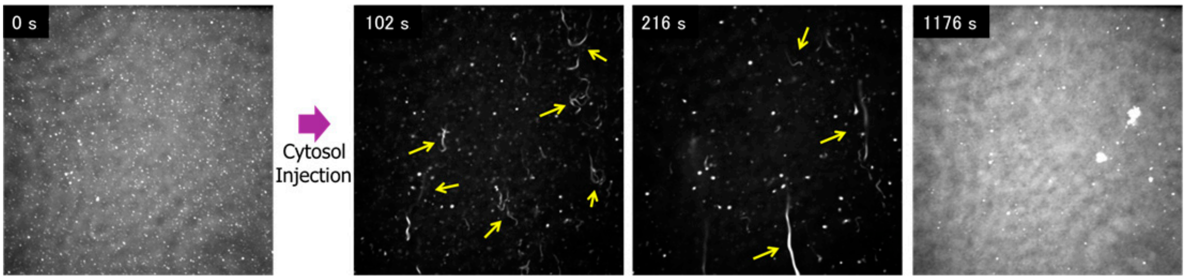

Figure S5. Time-course change of the fluorescence images of POPC SLB membrane on the flat slide glass (not using bottom open dish) after injection of the cytosol. Tubular giant vesicles on the SLB membrane were generated remarkably for initial ~300 s after cytosol injection (24–30 s). The SLB membrane was stained with TexasRed-DHPE. Yellow arrows show tGVs. The size of each observation space is 75.50 μm × 75.50 μm.
